# The Screening and Identification of Potential Probiotics in *Misgurnus anguillicaudatus* Intestine

**DOI:** 10.3390/ijms27209097

**Published:** 2026-10-14

**Authors:** Zhaoxin Sui, Mingshuai Shu, Ruining Zhang, Jianing Yu, Zhuo Dai, Yi Sun, Jun Li

**Affiliations:** 1 School of Life Science, Liaoning Normal University, Dalian 116029, China; suimoumourespect@163.com (Z.S.); 15504285774@163.com (M.S.); 18724308452@163.com (R.Z.); yjn621621@163.com (J.Y.); 18740176484@163.com (Z.D.); 2 Key Laboratory of Loach Genetics and Breeding of Jiangsu Province, Soochow University, Suzhou 215123, China; syixz524@suda.edu.cn; 3 School of Basic Medical Sciences, Suzhou Medical College of Soochow University, Suzhou 215123, China

**Keywords:** *Misgurnus anguillicaudatus*, intestinal microbiota, digestive enzymes, probiotics

## Abstract

We characterized the intestinal microbiota of *Misgurnus anguillicaudatus* using 16S rRNA gene sequencing (V4 region). Strains secreting extracellular digestive enzymes were functionally screened, identified by 16S rRNA sequencing, and evaluated for biosafety. Microbial diversity analysis revealed that the intestinal microbiota of *M. anguillicaudatus* was dominated by the genera *Vibrio*, *Pseudoalteromonas*, and *Aeromonas* as core predominant taxa. Functional screening yielded four strains. Three non-hemolytic strains secreting amylase, lipase, and protease were identified as *Aeromonas hydrophila*, *Shewanella xiamenensis*, and *Cedecea neteri*, respectively. *A. veronii* was excluded due to its β-hemolytic activity. Biosafety assessment indicated that all three strains exhibited satisfactory biosafety. The three strains, *A. hydrophila*, *S. xiamenensis*, and *C. neteri*, obtained in this study possess potential probiotic functions in aiding host digestion and promoting nutrient absorption, and provide candidate strains for the development of microecological preparations for aquatic animals.

## 1. Introduction

The Chinese loach (*Misgurnus anguillicaudatus*), belonging to the family Cobitidae is a freshwater benthic fish species inhabiting the bottom layers of rivers and lakes, with a wide distribution across major river systems in China. Loach is a freshwater fish of significant economic importance in Chinese aquaculture, with rapid growth and high market demand. Its meat is highly palatable, with a balanced amino acid profile, abundant fatty acids, and being rich in trace minerals, contributing to its nutritional and medicinal value for farmed populations [1]. According to the China Fishery Statistical Yearbook, the national production of loach reached 278,332 tons in 2024. *M. anguillicaudatus* holds important economic value in aquaculture, with expanding farming scale and rising market demand for its meat and nutritional profile. The species offers opportunities to improve growth performance, feed utilization, and disease resistance through gut microbiota modulation and functional enzyme-producing probiotics, contributing to more sustainable and productive aquaculture. Currently, aquaculture is developing toward intensification and high-density cultivation to increase unit water body yield and economic benefits to meet growing market demands; however, environmental stress and disease prevention and control issues have become increasingly prominent. Probiotics have received extensive attention from academia and industry as a management strategy for improving the production performance and health status of aquaculture animals through multiple pathways, including enhancing immune function, promoting digestion, resisting pathogenic bacteria, optimizing water quality, and facilitating growth, development, and reproduction [2]. To date, multiple probiotic species have demonstrated promising application potential in aquaculture, including *Lactobacillus*, *Lactococcus*, *Leuconostoc*, *Pseudomonas*, *Enterococcus*, *Carnobacterium*, *Shewanella*, *Bacillus*, *Aeromonas*, *Vibrio*, *Enterobacter*, *Clostridium*, *Pediococcus*, *Micrococcus*, and *Saccharomyces* [3,4,5,6,7,8]. It should be noted that there is limited scholarship regarding the application of opportunistic microorganisms as candidate probiotic strains. Most bacteria in aquatic environments are saprophytes and may display pathogenic traits under specific environmental conditions. Exoenzymes produced by these isolates may contribute to probiotic functions, yet they can also act as virulence-associated factors. Accordingly, only specific commensal strains within the genera *Vibrio* and *Aeromonas* can be regarded as potential probiotic candidates, rather than all members of these two genera. Notably, most *Aeromonas hydrophila* strains can exert virulence effects under adverse aquaculture conditions, raising the risk that strains with probiotic-related properties may turn into conditional pathogens. Therefore, sufficient safety evaluation is indispensable before such strains are developed as probiotics.

Given the above risks and the lack of host-derived probiotic resources for loach intensive culture, the present study aimed to screen intestinal bacteria from wild *M. anguillicaudatus* with extracellular enzyme-producing capacity and preliminarily evaluate their safety characteristics. This work expects to provide candidate strains and theoretical reference for developing host-specific probiotic preparations for loach healthy aquaculture.

## 2. Results

### 2.1. Analysis of Loach Intestinal Community Structure

**Figure 1:**
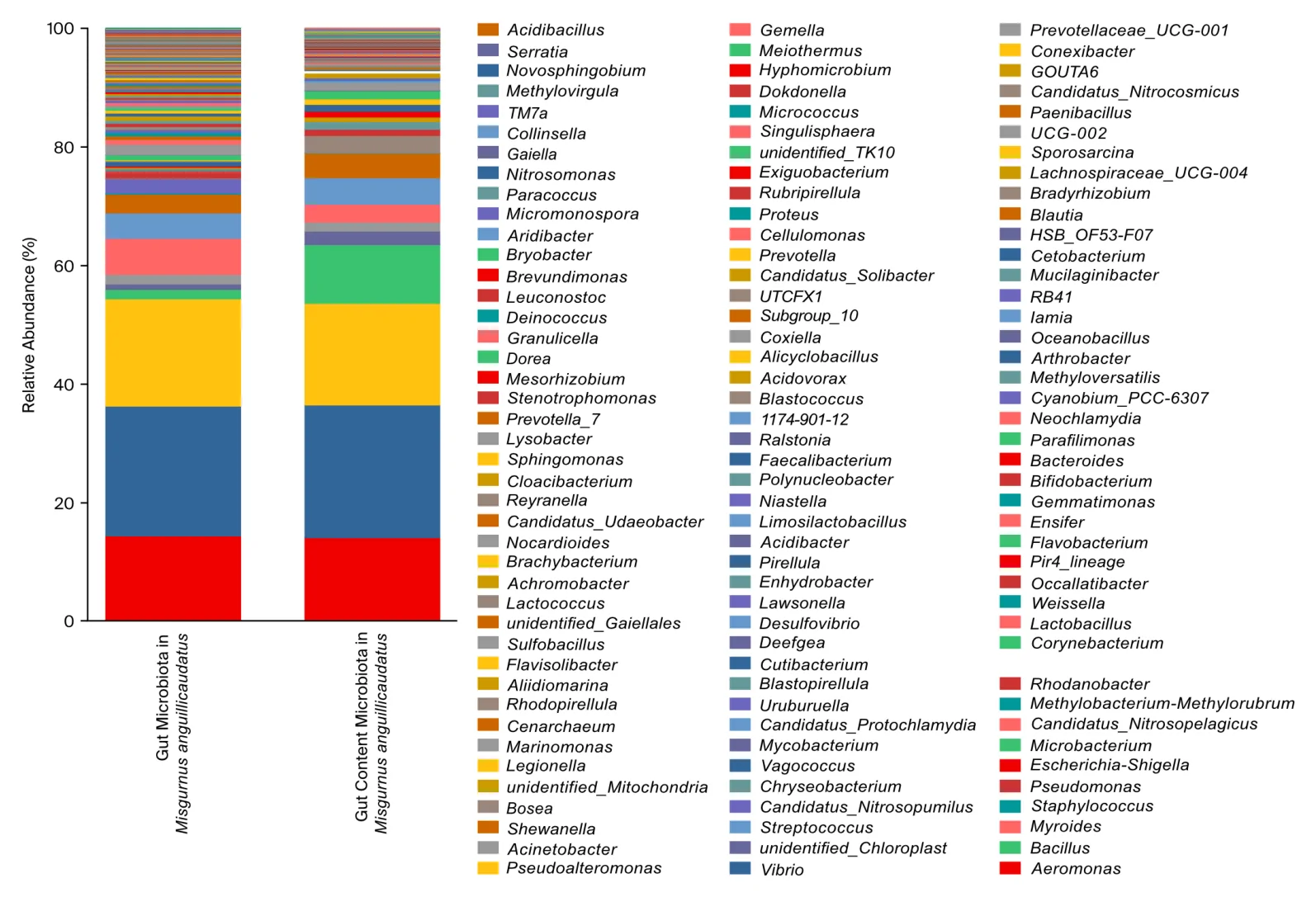
Stacked bar chart of relative abundance at the genus level of gut microbiota and gut content microbiota in *M. anguillicaudatus*.

As shown in Figure 1, the number of bacterial genera in loach intestinal contents (305) was higher than that in loach intestinal wall (293). *Vibrio* (21.87%), *Pseudoalteromonas* (18.07%), and *Aeromonas* (14.43%) were the core dominant genera in the loach intestinal microbiota, representing the major constituent groups. Similar to the intestinal microbiota, *Vibrio* (22.4%), *Pseudoalteromonas* (17.13%), *Aeromonas* (14.11%), and *Bacillus* (9.86%) exhibited relatively high abundances in the intestinal content microbiota, serving as the core dominant genera. In loach intestinal contents, the relative abundances of *Aeromonas*, *Shewanella*, and *Cedecea* were 14.11%, 4.09%, and 0.3%, respectively. The corresponding dataset can be found in the Supplementary Materials.

### 2.2. Prediction of Enzyme Functions in Loach Intestinal Microbiota

**Figure 2:**
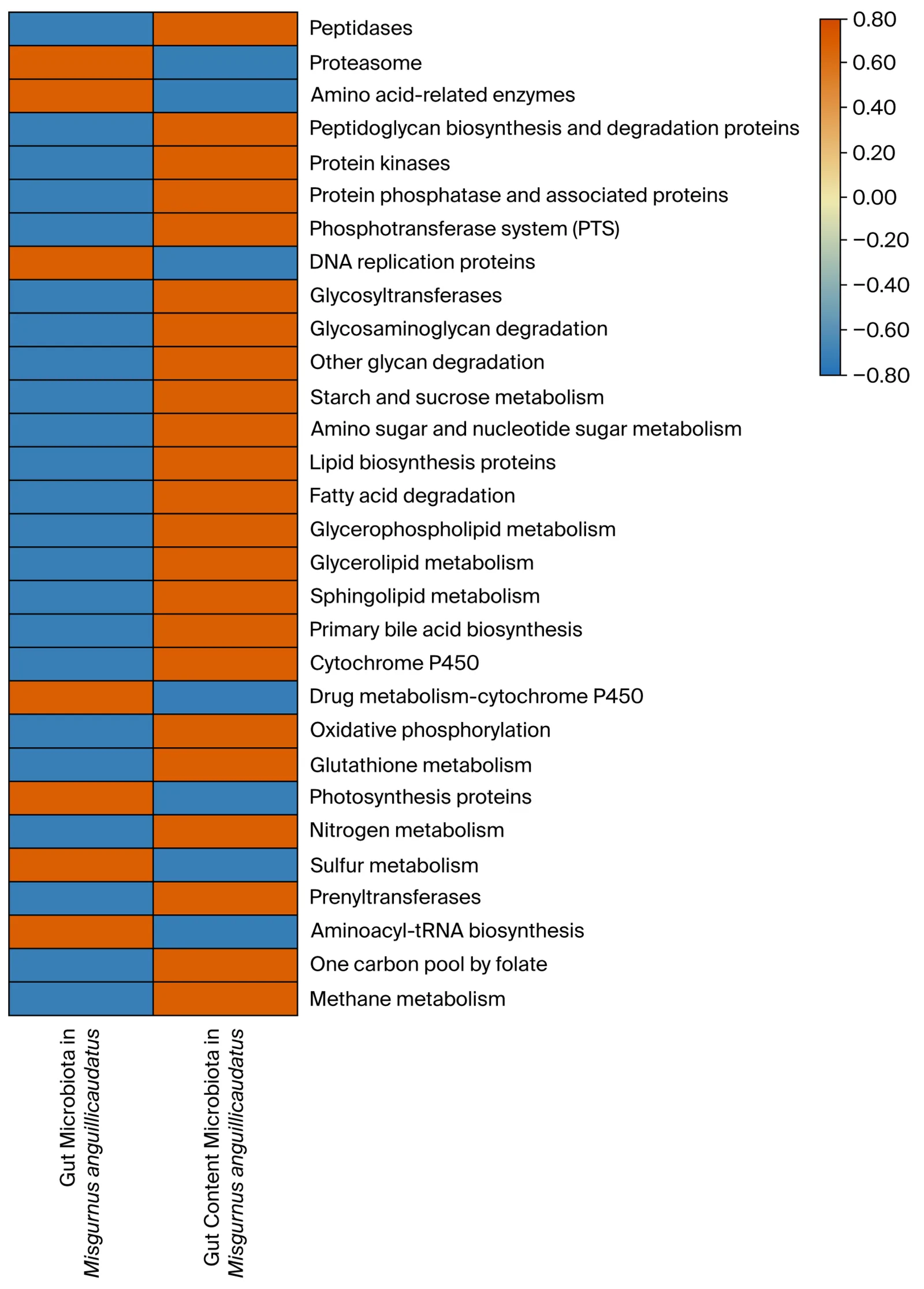
Cluster heatmap of level 3 enzyme family functions of intestinal microbiota in *M. anguillicaudatus* predicted by Tax4Fun.

As shown in Figure 2, compared with the intestinal microbiota, the level 3 metabolic functions of enzyme families in the intestinal content microbiota were significantly upregulated in protein, fatty acid, and carbohydrate synthesis and degradation. Enzyme families associated with protein degradation, modification, synthesis, and amino acid metabolism included peptidases, proteasome, amino acid-related enzymes, peptidoglycan biosynthesis and degradation, protein kinases, protein phosphatase and associated proteins, glutathione metabolism, nitrogen metabolism, sulfur metabolism, aminoacyl-tRNA biosynthesis, and one-carbon pool by folate. Those associated with polysaccharide degradation, sugar transport, glycosylation, and sugar nucleotide metabolism included phosphotransferase system (PTS), glycosyltransferases, glycosaminoglycan degradation, other glycan degradation, and starch and sucrose metabolism. Those associated with fatty acid, glycerophospholipid, sphingolipid, and isoprenoid metabolism included lipid biosynthesis proteins, fatty acid degradation, glycerophospholipid metabolism, glycerolipid metabolism, sphingolipid metabolism, primary bile acid biosynthesis, and prenyltransferases. Relevant data are provided in the Supplementary Materials.

### 2.3. Isolation and Identification of Enzyme-Producing Strains

Enzyme-producing capabilities were analyzed for strains isolated from the intestine of healthy wild loach, and their abilities to secrete amylase, lipase, and protease were determined (Table 1). Following 16S rRNA identification, *A. veronii* and *A. hydrophila* were identified as amylase-producing strains with Dh/Dc values of 1.70 and 1.43, respectively; *A. hydrophila* and *S. xiamenensis* as protease-producing strains with Dh/Dc values of 2.82 and 2.46, respectively; and *C. neteri* as a lipase-producing strain with a Dh/Dc value of 3.0. To verify whether the isolated strains possessed the ability to decompose the other two nutrient types, strains were inoculated onto the other two media. Cross-decomposition testing revealed that *A. veronii* and *A. hydrophila* could simultaneously decompose starch and protein, *S. xiamenensis* could simultaneously decompose protein and lipid, while *C. neteri* showed no cross-decomposition capability and only produced lipase.

### 2.4. Antimicrobial Susceptibility Testing

As shown in Table 2, among *Aeromonas* strains, *A. veronii* was susceptible to oxytetracycline, florfenicol, ampicillin, and kanamycin, whereas *A. hydrophila* was susceptible to oxytetracycline, ampicillin, and kanamycin, but intermediate to florfenicol. *S. xiamenensis* was susceptible to oxytetracycline, ampicillin, and kanamycin, and resistant to florfenicol. *C. neteri* was susceptible to oxytetracycline, ampicillin, and kanamycin, but resistant to florfenicol.

### 2.5. Hemolytic Assay

**Figure 3:**
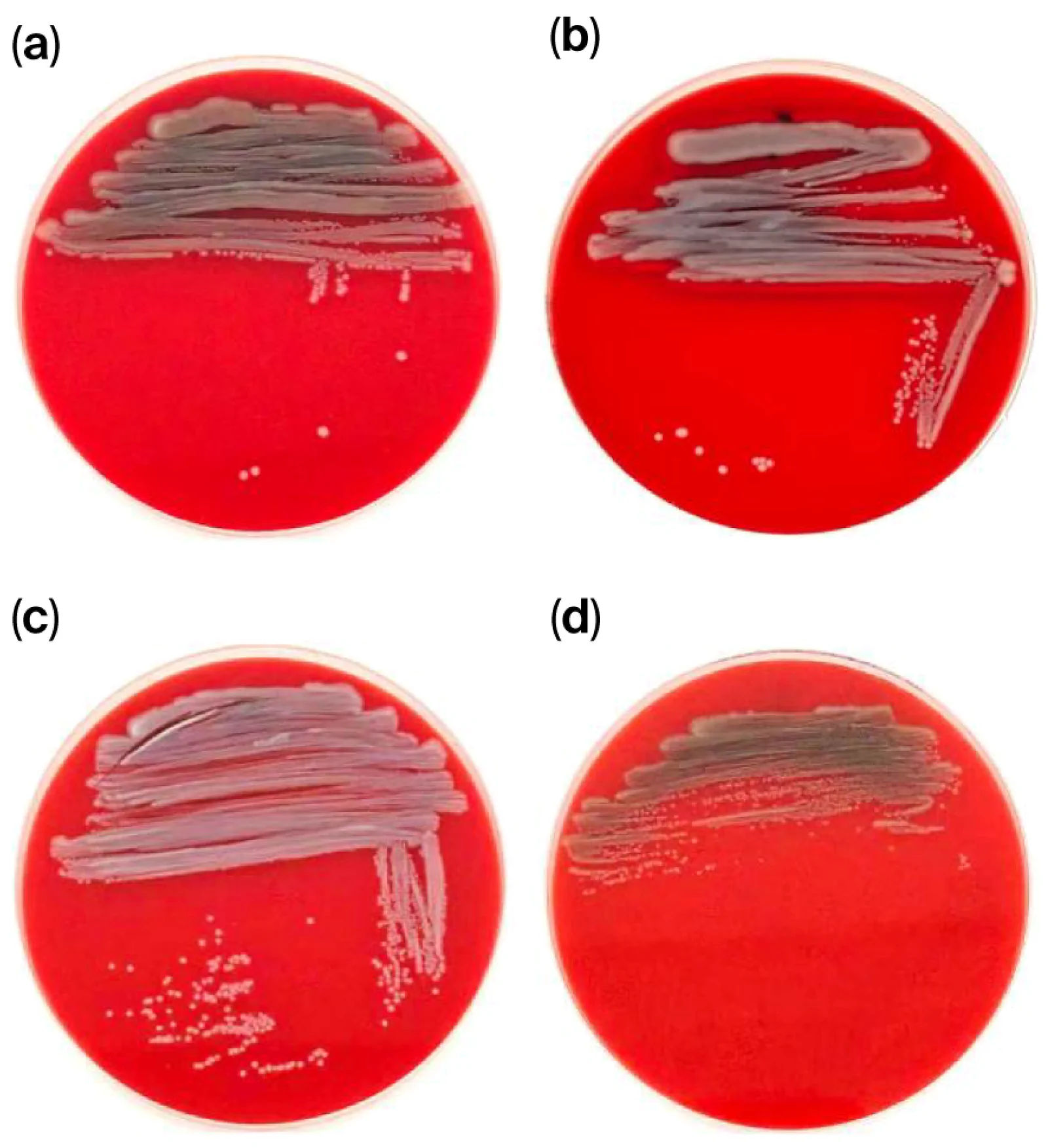
Hemolytic assay results of the strains. (**a**). Hemolytic assay result of *A. veronii*; (**b**). hemolytic assay result of *A. hydrophila*; (**c**). hemolytic assay result of *C. neteri*; (**d**). hemolytic assay result of *S. xiamenensis*.

Hemolytic assay results showed that *A. veronii* produced a clear and transparent hemolytic ring on Columbia blood agar, which exhibited typical β-hemolytic characteristics, indicating that this strain could produce hemolysin and lyse erythrocytes, possessing strong hemolytic activity and suggesting certain virulence potential. In contrast, *A. hydrophila*, *S. xiamenensis*, and *C. neteri* showed no obvious hemolytic phenomenon under the same culture conditions, with no hemolytic ring formation around colonies, and were considered non-hemolytic, indicating that these three strains had low expression levels of hemolysis-related virulence factors and relatively favorable biosafety (Figure 3).

### 2.6. Safety Assessment

Healthy loach were selected; the experimental groups were injected intraperitoneally with 0.1 mL live bacterial suspensions (10^6^ CFU·mL^−1^, 10^7^ CFU·mL^−1^, and 10^8^ CFU·mL^−1^) of *A. hydrophila*, *S. xiamenensis*, and *C. neteri*, respectively, and the control group was injected with an equal volume of physiological saline. After 14 d of rearing, results showed that loach in both experimental and control groups remained in good condition, with no mortality or lesions observed during the experimental period, indicating that *A. hydrophila*, *S. xiamenensis*, and *C. neteri* exhibited favorable safety to juvenile loach. *A. veronii* was not subjected to safety assessment due to its demonstrated hemolytic toxicity.

## 3. Discussion

Extracellular digestive enzymes synthesized by microorganisms in the digestive tract are considered key factors assisting host substance metabolism, significantly enhancing host digestive efficiency and growth performance [8,9,10]. The animal digestive tract represents a potential source of enzyme-producing microorganisms, and digestive tract-derived microbes exhibit stronger niche adaptability and colonization capacity within the host intestinal environment. Consequently, screening potential probiotics with extracellular enzyme secretion capabilities from the intestines of healthy animals has become a common strategy for probiotic acquisition [11]. Previous studies have demonstrated that digestive tract-derived microorganisms can produce amylase, protease, and lipase [12,13,14]; these three enzyme categories represent key functional enzymes in fish digestive physiology, playing central roles in the decomposition and metabolic transformation of carbohydrates, proteins, and lipids in feed [12,13,15]. The exploitation and utilization of digestive enzyme-producing microorganisms from the digestive tracts of healthy animals provide an alternative solution for optimizing feed costs and enhancing aquaculture production [16].

In this study, four bacterial strains with extracellular enzyme secretion capabilities were isolated from loach intestine and identified as *A. veronii*, *A. hydrophila*, *S. xiamenensis*, and *C. neteri*. *Aeromonas* belongs to the family Aeromonadaceae, capable of secreting multiple enzyme types [17]; its pathogenicity is closely associated with extracellular products [18]. Zeng et al. [19] investigated *Aeromonas* in the intestine of healthy grass carp (*Ctenopharyngodon idella*), isolating 76 Aeromonas strains, of which 50 secreted amylase and 49 secreted protease; all strains exhibited non-pathogenicity upon hemolysin detection. In this study, two *Aeromonas strains* secreting extracellular enzymes were isolated from loach intestine, namely *A. veronii* and *A. hydrophila*. Among them, *A. hydrophila* possessed the ability to secrete amylase and protease, and exhibited biosafety. Consistent with the results of this study, Zhu et al. [20] isolated 19 *A. hydrophila* strains from the intestines of common carp (*Cyprinus carpio*), grass carp (*Ctenopharyngodon idella*), silver carp (*Hypophthalmichthys molitrix*), and blunt snout bream (*Megalobrama amblycephala*). These strains did not produce hemolysin but could secrete extracellular enzymes, promoting fish growth. Although *Aeromonas hydrophila* is generally considered an opportunistic pathogen of aquatic animals, extensive studies have demonstrated that non-hemolytic, intestinal-derived strains exhibit no pathogenicity to healthy hosts when intestinal microecological balance is maintained [21]. Instead, these strains can assist host digestion, improve feed utilization efficiency, and exert positive effects on aquatic animal growth [20]. The key distinction lies in the hemolytic phenotype and virulence gene profile: while pathogenic strains typically exhibit β-hemolysis and carry multiple virulence genes, the A. hydrophila strain isolated in this study showed no hemolytic activity and passed all biosafety assessments, supporting its potential as a safe probiotic candidate. In this study, *A. veronii* exhibited typical β-hemolytic phenotype on blood agar, demonstrating definitive cytotoxicity. Previous studies have demonstrated that *A. veronii* represents an important opportunistic pathogen in various aquatic animals [22,23]. Li et al. [24] isolated 87 *A. veronii* strains from the intestines of healthy crucian carp (*Carassius auratus*), common carp (*Cyprinus carpio*), grass carp (*Ctenopharyngodon idella*), and catfish (*Silurus asotus*). The study found that nearly 50% of *A. veronii* carried at least four or more virulence genes, 25% carried at least five virulence genes, and the number of virulence genes carried was positively correlated with strain virulence. Nevertheless, it is crucial to emphasize that the pathogenicity of *Aeromonas* is strictly strain-specific, rather than a universal genus-level trait. For example, in Zhang et al. [25], strain D19 (*A. hydrophila*) showed no detectable virulence in challenge assays yet retained multiple virulence genes. Similarly, virulence among Aeromonas isolates appears to exist along a continuum rather than as a strict pathogenic or non-pathogenic dichotomy: Azzam-Sayuti et al. [26] reported LD50 values at 240 h ranging from 10^5^ CFU·mL^−1^ for *A. dhakensis* 4PS2 and *A. hydrophila* 8TK3 to 10^11^ CFU·mL^−1^ for *A. jandaei* 7KL3, indicating markedly attenuated but not absent virulence. Some isolates from healthy fish act as non-pathogenic commensals, while virulent isolates may trigger fatal septicemia in farmed fish [27,28]. Although most environmental *Aeromonas* strains are conditional pathogens, intestinal commensal strains isolated from healthy fish often lose key virulent factors and retain beneficial metabolic functions [25,29]. In the present study, the screened *A. hydrophila* strain lacked hemolytic activity and exhibited favorable extracellular enzyme-secreting ability, indicating that such endogenous intestinal strains are safe and possess probiotic potential rather than pathogenic risk. Therefore, strictly screened, non-pathogenic *Aeromonas* isolates from healthy fish intestines can be rationally developed as promising probiotic candidates for aquaculture application.

*Shewanella* constitutes a component of the gastrointestinal microbiota in naturally cultured fish [30]. As fish probiotics, *Shewanella* exerts positive effects in promoting growth [31,32,33,34], optimizing intestinal microbiota [35,36], regulating immunity [37,38,39], and enhancing stress resistance [30]. Previous studies have demonstrated that *Shewanella* can secrete protease [40,41] and lipase [42,43,44]. This study further confirmed that *S. xiamenensis* possesses lipase and protease secretion activities and exhibits biosafety. Meanwhile, studies have demonstrated that *S. xiamenensis* can effectively improve the intestinal microbial community structure of grass carp [36,38], reduce the relative abundance of potential pathogens (such as *Pseudomonas* and *Flavobacterium*), and promote the proliferation of potential probiotics (such as *Streptococcus* and *Enterococcus*). Additionally, *S. xiamenensis* can enhance innate immunity and disease resistance in grass carp, representing an intestinal-derived candidate probiotic with dual functions of microbiota regulation and immune modulation.

*Cedecea neteri* belongs to the family Enterobacteriaceae and genus *Cedecea*, and positive lipase activity represents one of the typical biochemical characteristics of this genus [45]. In this study, one *C. neteri* strain was screened from loach intestinal contents, which exhibited lipase secretion capability. Antimicrobial susceptibility testing, hemolytic assay, and host safety assessment confirmed that this strain safe for the loach host and devoid of pathogenic effects.

## 4. Materials and Methods

### 4.1. Experimental Materials

Healthy loach (*M. anguillicaudatus*) were purchased from the Greater Khingan Mountains region, Heilongjiang Province, without acclimation to artificial feed. A total of 156 individuals were used, with a body weight of 15.02 ± 2.01 g and a total length of 15.97 ± 1.32cm. Among them: 30 individuals were sampled for 16S rRNA high-throughput sequencing; intestinal contents from another 6 individuals were collected for isolation and screening of intestinal bacteria; the remaining 120 individuals were applied for the in vivo biosafety assay.

### 4.2. Experimental Reagents and Culture Media

Gentamicin and penicillin were purchased from North China Pharmaceutical Group Corporation (Shijiazhuang, China); oxytetracycline and florfenicol were purchased from Huamukang Animal Pharmaceutical Co., Ltd. (Manaus, Brazil); ampicillin and kanamycin were purchased from Brazilian Institute of Biotechnology and Innovation; Columbia blood agar was purchased from Guangdong Huankai Microbial Sci. & Tech. Co., Ltd. (Guangzhou, China); Lugol’s iodine solution was purchased from Lanbao Medical & Health Products Co., Ltd. (Shanghai, China); casein and soluble starch were purchased from Shanghai Sangon Biological Engineering Technology & Services Co., Ltd. (Shanghai, China).

Beef extract peptone medium: beef extract 5 g·L^−1^, peptone 10 g·L^−1^, NaCl 5 g·L^−1^, agar 16 g·L^−1^, pH 7.2–7.4. Protein medium—Solution A: peptone 13.33 g·L^−1^, yeast extract 4 g·L^−1^, NaCl 6.67 g·L^−1^, agar 21.33 g·L^−1^; Solution B: casein 40 g·L^−1^. Starch medium: beef extract 3 g·L^−1^, peptone 10 g·L^−1^, starch 10 g·L^−1^, NaCl 5 g·L^−1^, agar 16 g·L^−1^, pH 7.2–7.4. Lipid medium: peptone 10 g·L^−1^, yeast extract 5 g·L^−1^, CaCl_2_·H_2_O 0.1 g·L^−1^, Tween-80 10 mL·L^−1^, agar 16 g·L^−1^, pH 7.2–7.4.

### 4.3. Experimental Methods

#### 4.3.1. Sample Collection

Healthy loach were selected and euthanized by anesthesia with MS-222 (100 mg·L^−1^), then placed on clean bench. The body surface was disinfected with 75% ethanol, and the abdominal cavity was opened with sterile dissecting scissors to remove the intestine into a sterile Petri dish. After rinsing the intestine with sterile physiological saline (0.85%), the intestinal contents and intestinal wall tissues were separately collected into sterile cryovials, snap-frozen in liquid nitrogen, and stored at −80 °C. Intestinal contents and intestinal wall tissues were sampled separately. For each tissue type, samples harvested from six loaches were pooled to form one composite sample, and six biological replicates were prepared respectively. One aliquot was used for loach gut microbiota screening experiments, and the other five were frozen for subsequent DNA extraction.

#### 4.3.2. Analysis of Microbiota Characteristics in Intestinal Contents and Intestinal Wall

Total genomic DNA was extracted from loach intestinal contents and loach intestinal wall using a bacterial genomic DNA extraction kit. The V4 hypervariable region of the bacterial 16S rRNA gene was amplified using the specific primers 515F (5′-GTGCCAGCMGCCGCGGTAA-3′) and 806R (5′-GGACTACHVGGGTWTCTAAT-3′). The PCR products were purified, pooled in equimolar ratios, ligated with sequencing adapters, and used for sequencing library construction. High-throughput sequencing was subsequently performed on the Illumina NovaSeq 6000 platform (Illumina, Inc., San Diego, CA, USA).

The raw sequencing data were assembled and filtered to obtain high-quality clean tags. Tags were aligned against the Silva database (https://www.arb-silva.de (accessed on 25 September 2026)) to generate effective tags. The DADA2 module in QIIME2 (version QIIME2-202202) was used to obtain amplicon sequence variants (ASVs), and QIIME2 was employed for species annotation (Silva 138.1 database). Species abundance tables at phylum, class, order, family, genus, and species levels were generated based on ASV annotation results and sample feature tables. Tax4Fun was used to analyze metabolic activities of the microbiota, and functional prediction of loach intestinal content and intestinal wall microbiota was performed according to the Kyoto Encyclopedia of Genes and Genomes (KEGG, SILVA_123_SSURef_Nr99_tax_silva) database.

#### 4.3.3. Isolation and Purification of Strains

Under aseptic conditions, 0.1 g of intestinal contents was transferred into a sterile centrifuge tube containing 5 mL of pre-cooled sterile physiological saline (pH 7.2) and thoroughly vortexed to prepare the stock solution. The stock solution was serially diluted to 10^−3^ CFU·mL^−1^, 10^−4^ CFU·mL^−1^, and 10^−5^ CFU·mL^−1^. (Note: The exact CFU concentration of the original stock solution was not determined in this study; bacterial enumeration was performed based on colony counts from the diluted samples). Aliquots of 0.1 mL from each dilution (10^−3^ CFU·mL^−1^, 10^−4^ CFU·mL^−1^, and 10^−5^ CFU·mL^−1^) were inoculated onto beef extract peptone medium. Three replicates were established for each dilution, and incubated at 28 °C for 24–48 h. Single colonies were selected and streaked on plates for three consecutive generations to achieve purification. A total of 110 bacterial strains were isolated. Seven distinct strains were selected for purification and culture based on differences in colony size, color, texture, edge morphology and other morphological characteristics.

#### 4.3.4. Screening of Enzyme-Producing Strains

Purified strains were suspended in 200 µL sterile physiological saline to prepare stock solutions. The stock solutions were serially diluted to 10^−3^ CFU·mL^−1^, 10^−4^ CFU·mL^−1^, and 10^−5^ CFU·mL^−1^, and 0.1 mL aliquots were spread onto protein medium, starch medium, and lipid medium, respectively. Three replicates were established for each dilution and incubated at 28 °C for 24–48 h. Hydrolysis capacity (HC), defined as the ratio of hydrolysis zone diameter (Dh) to colony diameter (Dc) (HC = Dh/Dc), was used to indicate the organic matter decomposition ability of strains. Lipid and protein hydrolysis abilities were determined by direct observation of transparent hydrolysis zones surrounding colonies. Starch hydrolysis ability was assessed by adding an appropriate amount of Lugol’s iodine solution onto the medium surface, standing for 1–2 min, discarding the iodine solution, and immediate observation.

#### 4.3.5. Species Identification of Enzyme-Producing Strains

Genomic DNA of enzyme-producing strains was extracted using the Bacterial Genomic DNA Extraction Kit (Sangon Biotech B518255, Shanghai, China). The full-length 16S rRNA gene (expected amplicon size: ~1465 bp) was amplified using universal primers 27F (5′-AGAGTTTGATCMTGGCTCAG-3′) and 1492R (5′-TACGGYTACCTTGTTACGACTT-3′). PCR products with correct band sizes were sequenced by Shanghai Sangon Biological Engineering Technology & Services Co., Ltd. The obtained sequences were subjected to BLAST analysis on NCBI (https://blast.ncbi.nlm.nih.gov/Blast.cgi (accessed on 25 September 2026)), and phylogenetic trees were constructed using MEGA 11 software. Species identification was determined based on a gene sequence similarity criterion of ≥99%. The four 16S rRNA gene sequences generated in this study have been deposited in the GenBank database under accession numbers PZ734422 (*Aeromonas hydrophila*), PZ734423 (*Aeromonas veronii*), PZ734424 (*Shewanella xiamenensis*), and PZ734425 (*Cedecea neteri*), respectively.

#### 4.3.6. Antimicrobial Resistance Characteristics of Enzyme-Producing Strains

Six antibiotics commonly used in aquaculture (oxytetracycline hydrochloride, gentamicin, penicillin, florfenicol, ampicillin, and kanamycin) were selected for antimicrobial susceptibility testing by the disk diffusion method. Purified enzyme-producing strains were inoculated into LB liquid medium and cultured at 28 °C, 220 r·min^−1^ to logarithmic growth phase (OD_600_ = 1). One milliliter of bacterial suspension was centrifuged at 4 °C, 8000 r·min^−1^ for 5 min; the supernatant was discarded; and the pellet was washed three times with sterile physiological saline and resuspended to adjust the bacterial concentration to OD_600_ = 0.1. One hundred microliters of bacterial suspension was evenly spread onto LB solid medium and stood at room temperature for 5 min. Antimicrobial disks impregnated with oxytetracycline hydrochloride (30 µg/disk), gentamicin (10 µg/disk), penicillin (10 µg/disk), florfenicol (30 µg/disk), ampicillin (10 µg/disk), and kanamycin (30 µg/disk) were placed on the medium surface inoculated with enzyme-producing strains, with 4–6 disks per plate, spacing ≥ 24 mm, and distance from edge ≥ 15 mm. After incubation at 28 °C for 10 h, the inhibition zone diameter (mm, including disk diameter) was measured and observed. Results were classified as susceptible (S), intermediate (I), or resistant (R) according to standards established by the CLSI (Clinical and Laboratory Standards Institute) and the EUCAST (European Committee on Antimicrobial Susceptibility Testing), combined with bacterial species, antimicrobial agent type, culture medium, and test conditions (e.g., disk diffusion method at 28 °C for 10 h). The specific inhibition zone diameter breakpoints for each antimicrobial agent were as follows: oxytetracycline (30 µg/disk): R ≤ 13 mm, I 14–18 mm, S ≥ 19 mm; florfenicol (30 µg/disk): R ≤ 12 mm, I 13–17 mm, S ≥ 18 mm; ampicillin (10 µg/disk): R ≤ 13 mm, I 14–16 mm, S ≥ 17 mm; kanamycin (30 µg/disk): R ≤ 13 mm, I 14–17 mm, S ≥ 18 mm.

#### 4.3.7. Hemolytic Assay of Enzyme-Producing Strains

Enzyme-producing strains were inoculated into LB liquid medium and cultured at 28 °C, 220 r·min^−1^ to logarithmic growth phase, then streaked onto Columbia blood agar medium and incubated at 28 °C in inverted position for 24–48 h to observe whether a transparent hemolytic zone appeared around the colonies.

#### 4.3.8. Safety Assessment of Enzyme-Producing Strains

Enzyme-producing strains were inoculated into 400 mL liquid medium and cultured at 28 °C, 220 r·min^−1^ to the logarithmic growth phase (OD_600_ = 1). One milliliter of bacterial suspension was centrifuged at 4 °C, 8000 r·min^−1^ for 5 min; the supernatant was discarded; and the pellet was washed three times with sterile physiological saline and resuspended to adjust the bacterial concentration to 10^6^ CFU·mL^−1^, 10^7^ CFU·mL^−1^, and 10^8^ CFU·mL^−1^.

Healthy loach were randomly divided into four groups (three experimental groups and one control group), with 30 individuals per group and three replicates (10 individuals per replicate). Intraperitoneal injection was performed with 0.1 mL bacterial suspension per individual (control group injected with 0.1 mL sterile physiological saline) at inoculation doses of 10^5^, 10^6^, and 10^7^ CFU per individual. The fish were reared for 14 d, and mortality was recorded daily to evaluate the safety of potential probiotic strains to the host.

## 5. Conclusions

In this study, four bacterial strains with extracellular enzyme secretion capabilities were initially isolated from loach intestine, but one (*A. veronii*) was excluded due to β-hemolytic activity. The remaining three strains, identified as *A. hydrophila*, *S. xiamenensis*, and *C. neteri*, were preliminarily confirmed as safe candidate probiotics with amylase, protease, and lipase activities. These strains possess potential probiotic functions in assisting host digestion and promoting nutrient absorption, indicating their potential for development and application as probiotics for fish and other aquatic animals. However, further systematic evaluation through feeding experiments is required to verify probiotic efficacy, including growth promotion effects, disease resistance, long-term colonization capacity, and optimal formulation as single or composite agents.

## Figures and Tables

**Table 1:** Enzyme production capacity of intestinal bacteria in *M. anguillicaudatus*.

Enzyme-Producing Category	Hydrolysis Zone of Strains			
	*A. veronii*	*A. hydrophila*	*S. xiamenensis*	*C. neteri*
Amylase (Dh/Dc)	1.70	1.43	-	-
Lipase (Dh/Dc)	-	-	1.41	3.0
Protease (Dh/Dc)	2.41	2.82	2.46	-

Note: Dh/Dc represents the ratio of hydrolysis zone diameter (Dh) to colony diameter (Dc).

**Table 2:** Antibacterial activity of the strains.

Antimicrobial Agent		Dosage (µg)	Inhibition Zone Diameter Criteria (mm)			
			*A. veronii*	*A. hydrophila*	*S. xiamenensis*	*C. neteri*
Tetracyclines	Oxytetracycline	30	23.5/S	37/S	20/S	21/S
Chloramphenicols	Florfenicol	30	20/S	16/I	8/R	9/R
	Ampicillin	10	21/S	20/S	37/S	18/S
Aminoglycosides	Kanamycin	30	25/S	25/S	36/S	28/S

Note: R, resistant; I, intermediate; S, susceptible. Classification criteria are described in Section 4.3.6. Penicillin was not tested due to intrinsic resistance of aquatic bacteria to this agent.

## Data Availability

The original contributions presented in this study are included in the article/Supplementary Materials. Further inquiries can be directed to the corresponding author.

## Supplementary Materials

The following supporting information can be downloaded at: https://www.mdpi.com/article/10.3390/ijms27209097/s1.
